# Neurological Manifestations of Aluminum Phosphide (Wheat-Pill/Rice-Pill) Poisoning: A Narrative Review

**DOI:** 10.7759/cureus.98910

**Published:** 2025-12-10

**Authors:** Amir Rasheed, Syed Haider Hassan, Haseeb Mehmood Qadri, Abu-Bakr Ahmed, Muhammad Ahmed, Haysum Khan, Nusrat Fatima, Adeena Azam, Saad Abdullah Dar, Arham Amir Khawaja

**Affiliations:** 1 Internal Medicine, Aziz Bhatti Shaheed Teaching Hospital, Gujrat, PAK; 2 Neurological Surgery, University of Wisconsin, Madison, USA; 3 Neurological Surgery, Punjab Institute of Neurosciences, Lahore, PAK; 4 General Surgery, Lahore General Hospital, Lahore, PAK; 5 School of Medicine, University of Nevada Las Vegas School of Medicine, Las Vegas, USA; 6 Critical Care, Bahria International Hospital, Lahore, PAK; 7 Neurological Surgery, Shifa Tameer-e-Millat University, Shifa College of Medicine, Islamabad, PAK; 8 Respiratory Medicine, Portsmouth University Hospital NHS Trust, Portsmouth, GBR; 9 Internal Medicine, Allama Iqbal Medical College, Lahore, PAK; 10 Surgery, Allama Iqbal Medical College, Lahore, PAK; 11 General Surgery and Surgical Oncology, Shaikh Zayed Medical Complex, Lahore, PAK

**Keywords:** aluminum phosphide, cns, insecticide, low to middle income countries, nausea, neurological, rice pill, rodenticide, vomiting, wheat pill

## Abstract

The clinical manifestations of Aluminium Phosphide (AlP) that stem from its misdirected human consumption range from nausea and vomiting to acute respiratory failure, cardiotoxicity and hepatotoxicity, causing sudden death. Currently, there is no standard regimen to deal with this menacing product. This review aimed to highlight the neurological manifestations and treatment options for dealing with wheat pill poisoning.

After the literature search, a total of eight studies were included in this study. Study types included five case reports, a prospective case series, a retrospective review, and an autopsy with sample sizes ranging from a single patient to 471 patients. The major symptoms included were dizziness, headache, and weakness in both upper and lower extremities. Reported neurological findings included progressive decline in consciousness, anisocoria with non-reactive pupils, loss of consciousness, convulsions, variable coma grades, and motor deficits. The CT brain findings mentioned in one case report were significant for diffuse bilateral hypoattenuation in the cerebellar hemispheres, midbrain, thalamus, and globus pallidus nuclei. MRI brain revealed multiple cortical and subcortical diffusion restrictions in cerebral hemispheres, consistent with prior global hypoperfusion injury in one case report, while the other showed an acute ischemic infarct in the left posterior cerebral artery (PCA) territory involving left medial temporal, parieto-occipital lobes, left half of the splenium of the corpus callosum and left thalamus.

There is some evidence that paraffin oil, co-enzyme Q10, hyperinsulinemia euglycemia, and N-acetylcysteine (NAC) decrease mortality in this poisoning. However, further large-scale randomised controlled trials are needed to definitively evaluate their mortality benefit.

## Introduction and background

Aluminium phosphide (AlP) is used as a "wheat pill" or "rice pill" with rodenticide effects. However, it has been misused for poisoning. The clinical manifestations of AlP range from nausea and vomiting to acute respiratory failure, cardiotoxicity and hepatotoxicity, causing sudden death. Currently, there is no standard regimen to deal with this menacing product of household use [1].

In 1998, the World Health Organization (WHO) recognised acute pesticide poisoning as a growing health concern because it caused one million accidental poisonings annually, resulting in 20,000 fatalities and one million cases of intentional poisoning [2]. Metallic phosphides are one of the deadliest poisons used as pesticides and are categorised as Category I poisons by the United States Environmental Protection Agency. Due to their low cost and easy availability, they are widely used as pesticides in agriculture in resource-limited countries, which explains the high incidence of poisoning cases in these areas. Among the metallic phosphides, AlP and zinc phosphide account for most cases of poisoning in humans. They have very high mortality, ranging from 40% to 90% for AlP. Recent literature suggests an alarming increase in the rate of phosphide-related mortality with suicidal intent [2,3].

This review aimed to highlight the neurological manifestations and latest developments in dealing with wheat pill poisoning.

## Review

Mechanism of toxicity

AlP is commonly dispensed as wheat pills or rice pills. They are either inhaled unintentionally or ingested with suicidal intent. On coming in contact with moisture, AlP forms phosphine gas (PH3), the main culprit. After being formed, it readily diffuses into tissues and exerts its cytotoxic effects by interacting with complex IV of the inner membrane of mitochondria. In addition, it also inhibits cytochrome c oxidase and oxidative phosphorylation, resulting in failure of cellular respiration [2,3]. This failure of respiration at the cellular level has additional but very serious consequences, such as failure of ATP formation and generation of reactive oxygen species (ROS). Furthermore, there are additional hypotheses that it may also exert its toxic effects by inhibiting acetylcholine esterase and altering acetylcholine signaling. Together, these are thought to play the toxigenic mechanism of action, responsible for such a high mortality rate [2].

Generalized symptomology

Following inhalation, it may present with garlic-like or rotten eggs odor breath, respiratory tract irritation and in severe cases, acute respiratory distress syndrome (ARDS). On the other hand, ingestion of metallic phosphides may cause symptoms of gastrointestinal tract (GI) irritation such as nausea, vomiting or upper GI bleed. The manifestations of toxicity are usually apparent within a few minutes of ingestion and include cardiovascular collapse, pulmonary toxicity and hepatotoxicity. PH3 induces electrolyte changes and pH disturbances that include hypokalemia, hyper or hypomagnesemia and metabolic acidosis. In the first 12-24 hours, most of the fatalities are caused by dysrhythmias and severe refractory cardiogenic shock. After 24 hours, ARDS respiratory failure, hepatic and renal failure are the main causes of death. Most AlP-intoxicated patients collapse rapidly, and adequate supportive measures are insufficient to save their lives [2].

Neurological manifestations (symptoms & signs)

After the literature search, a total of eight studies were included in this study. Study types included five case reports, a prospective case series, a retrospective review, and an autopsy with histopathology, with sample sizes ranging from a single patient in case reports to 471 patients in the retrospective review. The major symptoms reported included dizziness, headache, and weakness in both upper and lower extremities, while additional features such as memory difficulties, confusion, and sudden loss of consciousness were also noted. Reported neurological findings included progressive decline in Glasgow Coma Scale (GCS), anisocoria with non-reactive pupils, loss of consciousness with variable period of onset, convulsions in a few cases, variable coma grades, and motor deficits such as hemiplegia, quadriparesis, and facial weakness; some cases also showed transient visual impairment and reduced reflexes (Table 1).

**Table 1:** Neurological symptoms and signs of aluminum phosphide poisoning

Article	Study By	Study Type	Cohort/Sample Size	Neurological Symptoms	Neurological Signs
1.	Abadi et al., 2022 [4]	Case Report	1	Confusion, drowsiness	Progression of GCS: 6/15 to 9/15 to 5/15 to 3/15. Initial anisocoria, then mydriatic and non-reactive pupils.
2.	Khurana and Gupta, 2015 [5]	Prospective Case Series	50	Dizziness 26/50 (52%), headache 22/50 (44%)	Convulsions 4/50 (8%), Conscious till death: 2/38 deaths (5.26%) did not lose consciousness. Loss of consciousness timing: 0–2 hours: 5/38 deaths (13.89%) of patients lost consciousness very early. 2–4 hours: The largest group (17/38 deaths (44.44%) lost consciousness in this period. 4–6 hours: 11/38 deaths (27.78%) lost consciousness in this window.
3.	Kargar et al., 2023 [6]	Case Report	1	• Unable to speak for 2 days after extubation • Memory, calculation difficulties • Difficulties in walking and maintaining balance • Reduced ability to read messages on the smartphone on follow-up	After extubation: severe bilateral visual impairment (1/10 acuity, later improved to 9/10)
4.	Shadnia et al., 2009 [7]	Retrospective	471	Dizziness 145/471 (30.77%), agitation 72/471 (15.38%), headache 72/471 (15.38%)	Decrease of deep tendon reflex 145/471 (30.77%), dilated pupil 109/471 (23.1%), coma grade I 314/471 (66.67%), coma grade II 52/471 (11.11%), coma grade III 52/471 (11.11%), coma grade IV 52/471 (11.11%)
5.	Hussain et al., 2014 [8]	Case report	1	Progressive weakness in bilateral upper and lower extremities, difficulty in ambulating	Symmetric flaccid, areflexic quadriparesis (strength of 4/5 in upper limbs and 2/5 in lower limbs).
6.	Abedini et al., 2014 [9]	Case report	1	Sudden onset left side body weakness and difficulty in speaking	Left hemifacial paresis, left hemiplegia; absent left plantar reflex, NIHSS of 16
7.	Jindal et al., 2020 [10]	Case report	1	Drowsy, Right-sided weakness with tingling sensation	Reduced right upper and lower extremity power
8.	Kumar et al., 2022 [11]	Autopsy + histopathology	1	Pre‑mortem course: rapid loss of consciousness≈ 2 h after ingesting 3 AIP tablets	None

Neurological manifestations (radiological findings and blood/serum/CSF derangements)

Radiological findings were reported using non-contrast computerized tomography (CT) brain in two studies and magnetic resonance imaging (MRI) brain in three studies. The CT brain findings mentioned in one case report were significant for diffuse bilateral hypoattenuation in the cerebellar hemispheres, midbrain, thalamus, and globus pallidus nuclei. Tonsillar and transtentorial herniations, mild obstructive hydrocephalus were also observed. MRI brain revealed multiple cortical and subcortical diffusion restrictions in cerebral hemispheres, consistent with prior global hypoperfusion injury in one case report, while the other showed an acute ischemic infarct in the left posterior cerebral artery (PCA) territory involving left medial temporal, parieto-occipital lobes, the left half of the splenium of corpus callosum and left thalamus. While the autopsy revealed necrotic patches and oedema over cerebellar cortex on gross examination, and micro‑vascular congestion, neuronal necrosis, and depleted glia histopathology (Table 2).

**Table 2:** Radiological, blood/serum/CSF findings in aluminum phosphide poisoning ABG = Arterial blood gas, VBG = Venous Blood Gas, HCO₃⁻ = Bicarbonate, Cr = creatinine, AST = aspartate aminotransferase, ALT = alanine aminotransferase, ALP = Alkaline phosphatase, GGT = gamma-glutamyl transferase, INR = International normalized ratio, CRP = C-reactive protein, ESR = Erythrocyte sedimentation rate, MCA = right middle cerebral artery, PTT = partial thromboplastin time, PT = prothrombin time, CK = Creatine kinase, PCA = Posterior Cerebral Artery, NIHSS = National Institutes of Health Stroke Scale, CSF = Cerebrospinal fluid **was an autopsy study

Article	Study By	Radiological Investigations	Blood / Serum / CSF Findings
1.	Abadi et al., 2022 [4]	Non‑contrast brain CT: diffuse bilateral hypoattenuation in the cerebellar hemispheres, midbrain, thalamus, and globus pallidus nuclei. Tonsillar and transtentorial herniations, mild obstructive hydrocephalus.	Hct: 41.3% (slightly low), BUN: 26 mg/dL (high); Cr: 1.4 mg/dL (slightly high), direct bilirubin 0.4 mg/dL (borderline high), pH 7.44; pCO₂: 23 mm Hg (low), HCO₃⁻: 22 mEq/L (low). Initial respiratory alkalosis followed by severe metabolic acidosis (pH: 7.18, HCO₃ : 12.5 mEq/L)
2.	Khurana and Gupta, 2015 [5]	None	None
3.	Kargar et al., 2023 [6]	Brain MRI: Multiple cortical & subcortical diffusion restrictions in cerebral hemispheres → consistent with prior global hypoperfusion injury	Initial mild metabolic acidosis: pH 7.23, HCO₃ 21 mEq/L, pCO₂ 49.6 mmHg. Serial labs showed nadir pH 7.171; HCO₃ 19.3 mEq/L
4.	Shadnia et al., 2009 [7]	None	Blood pH 7.23 and HCO₃⁻ 12.7 mEq/L
5.	Hussain et al., 2014 [8]	None	WBC: 22×10^3^/μL, haemoglobin: 8.9 g/dL (normocytic) and platelets: 600×10^3^/μL. Blood smear: normocytic anaemia with neutrophilic leucocytosis and a left shift. Total bilirubin: 8.8 mg/dL (direct 6.6 mg/dL), ALT 49 U/L, ALP 49 U/L, GGT 261 U/L, albumin 3.3 g/dL, globulin 2.9 g/dL. INR: 1.6. procalcitonin 1.75 ng/mL, CRP 28 mg/L. ESR 4 mm/h. Blood, urine, sputum and cerebrospinal fluid (CSF) cultures were all negative. ABG: pH 7.14, Hg, HCO3 13 mEq/L, VBG: pH 7.14, HCO3 13.5 mEq/L, CSF: acellular with a high protein of 273 mg/dL, lactate of 4 mmol/L and normal glucose levels. Aluminum level was elevated at 86 μg/L.
6.	Abedini et al., 2014 [9]	CT Brain: Normal, MRI Brain: Ischemic lesions in the right MCA territory, Brain MRA: Stenosis of the right MCA stem.	Platelet Count (per mm^3^) 103000, Serum Magnesium (mg/dl) 2.4, HDL 22, ALT (U/L) 220, AST (U/L) 58, Urea (mg/dl) 37, CK (mU/ml) 1056
7.	Jindal et al., 2020 [10]	MRI Brain: Acute ischemic infarct in left PCA territory involving left medial temporal, parieto-occipital lobes, left half of splenium of corpus callosum and left thalamus with mild mass effect effacing overlying cortical sulci and adjacent lateral ventricle	WBC 16660 mm^3^, Triglyceride 226 mg/dl, ALT 47 U/L
8.	**Kumar et al., 2022 [11]	No ante‑mortem imaging. Gross autopsy: necrotic patches & edema over cerebellar cortex. Histology: micro‑vascular congestion, neuronal necrosis, depleted glia	Toxicology positive for AIP

Severe metabolic acidosis was a consistent feature, with pH values between 7.23 and 7.14 and bicarbonate as low as 12.7 mEq/L, occasionally preceded by respiratory alkalosis. One case demonstrated marked hyperbilirubinemia (total 8.8 mg/dL; direct 6.6 mg/dL), with liver function tests (LFTs) showing mild aspartate transaminase and alanine transaminase (AST/ALT) elevations, and renal function tests (RFTs) mildly raised. Elevated aluminium levels (86 μg/L) were also reported, and toxicology at autopsy was positive for AIP (Table 2).

Results & Descriptive Thematic Analysis

We reviewed eight papers that describe how aluminium phosphide poisoning affects the nervous system. Two of them were hospital-based retrospective cohort studies. One comes from Tehran and followed 471 patients, and the other comes from India and includes 50 patients [5,7]. The remaining six papers narrate individual patients and one autopsy, and they give the level of clinical detail that large series rarely include [4,6,8-11].

Both cohorts are retrospective series from tertiary centres where most ingestions were intentional. That setting tends to include sicker patients and higher early mortality [5,7]. The single case studies focus on notable neurologic presentations and include tests such as MRI, nerve conduction studies, and histology that the cohorts rarely report [4,6,8-11].

Presentation in Cohorts

In both retrospective cohort studies (n=521 combined), nonspecific symptoms were most common on arrival. Pooled across cohorts, dizziness was seen in roughly a third of patients and headache in about a fifth. The series reported by Khurana et al. described higher rates than the series reported by Shadnia et al. [5,7]. This likely reflects differences in the patients who reached care or in the way symptoms were recorded. On examination, the Shadnia group also noted agitation, dilated pupils, and decreased deep tendon reflexes, and a meaningful minority presented in coma, usually not the deepest grades [7]. In other words, the initial neurologic picture often mirrors the overall severity of poisoning.

CNS Phenotypes from Single-Patient Reports

The case reports in the literature present different neurologic syndromes which expand our understanding of the condition. Two patients who survived the toxic crisis developed delayed ischemic strokes after their initial survival. The first patient developed a right middle cerebral artery infarct according to Abedini et al., which showed stenosis during angiographic examination [9]. The second patient described by Jindal et al. suffered left posterior cerebral artery territory infarcts that resulted in sensory and motor deficits [10]. The patient in Kargar et al.'s case study developed cortical visual loss because of global hypoperfusion. MRI revealed cortical and subcortical diffusion restriction. The patient's vision recovered gradually during one year [6]. A fulminant case showed diffuse encephalopathy with transtentorial and tonsillar herniation and symmetric deep grey and posterior fossa changes on CT, which ended in death [4].

Beyond central nervous system pathology, peripheral nerve involvement has also been observed. In the report by Hussain et al., the patient developed an acute axonal sensorimotor polyneuropathy with albumin-cytologic dissociation [8]. At the histopathological level, the autopsy case documented cerebellar necrosis, cortical disorganisation, Purkinje cell injury, and microvascular congestion, which links the clinical picture to tissue-level injury [11].

Acid Base Status and Outcomes

The analysis of blood gases revealed that severe metabolic acidosis appeared in every case. The sickest patients showed pH values between 7.14 and 7.23 with bicarbonate levels at 12-13 mEq/L. Shadnia et al. demonstrate that blood pH levels directly affect patient survival rates. The study demonstrated that patients who had arterial pH values under 7.0 died, but patients with pH values at 7.35 or higher survived [7]. The research findings demonstrate how phosphine exposure damages mitochondria and heart function, and brain perfusion, which leads to these results.

Mortality

Reported mortality was not the same across settings: about 31% in the cohort studied by Shadnia et al. (2009) versus 76% in the series reported by Khurana et al. (2015) [5,7]. That gap likely reflects who reached the hospital, how quickly they were treated, and what care was available. Even so, several single-patient reports show survival with residual deficits once the early cardiogenic and metabolic storm settled, including survivors of delayed stroke and cortical visual syndromes [4,6,10].

Treatment and Management Protocols

There is no standard regimen or specific antidote available for AlP intoxication that could potentially reverse the pathology [2,3]. Supportive management is considered the mainstay of treatment, and the most common interventions are GI decontamination with oils, especially coconut or olive oil, magnesium sulfate infusion and hemodynamic support for hypotension. However, there is no consensus and clinically, there is a wide variety of regimens being used. In the literature, there are different interventions that include various therapies. Interventions include modalities that target the airway and breathing, circulation, decontamination, elimination, and therapies that counteract poison effects [2-10]. Modalities that focus on the airway and breathing include extracorporeal membrane oxygenation and hyperbaric oxygen. The drugs that are reportedly used for circulatory support include amiodarone, digoxin, hydrocortisone, levosimendan, lidocaine, milrinone, trimetazidine, vasopressin and hydroxy starch. Gastric decontamination agents include coconut oil, olive oil, paraffin oil, potassium permanganate, sodium bicarbonate and sweet almond oil. The elimination strategies are hemodialysis, hemofiltration, peritoneal dialysis, plasmapheresis and renal replacement therapy. Among options that counteract PH3 effects, notable ones are atropine, ascorbic acid (Vitamin C), exenatide, fresh packed red blood cell transfusion, hyperinsulinemia euglycemia (HIE), glucagon, magnesium, methylene blue, N-acetylcysteine (NAC), potassium, pralidoxime, tocopherol (Vit. E), thyroid hormones and whole blood transfusion [2]. Due to its high mortality, finding a cure is a dream of every clinician who manages these cases.

A recent review highlighted the important therapies that may have a lifesaving role in AIP poisoning [2]. Therapies that proved to reduce mortality were gastric decontamination with paraffin oil, HIE therapy, NAC and fresh RBC transfusion. A study reported a statistically significant reduction in mortality, from 73% to 36%, if GI decontamination was done with aspiration and paraffin oil left in the stomach. The addition of co-enzyme Q10 resulted in further reduction of mortality to an astonishing 23% [2,12]. Additionally, a clinical trial has evaluated the mortality benefit of HIE therapy and concluded that it is a safe and effective treatment, improving hemodynamic parameters and resulting in a statistically significant reduction in mortality from 96.3% to 63.4% as compared to the control group [13]. Another review summarised the use of NAC concluded that NAC not only resulted in a significant reduction of mortality and decreased length of stay in hospital but also increased survival duration among non-survivors [14].

The elimination strategies are hemodialysis, hemofiltration, peritoneal dialysis, plasmapheresis and renal replacement therapy. The efficacy of these strategies is still under consideration. A systematic review and meta-analysis on the use of extracorporeal membrane oxygenation (ECMO) suggested a decrease in mortality rate in AIP poisoning. However, despite its promise, ECMO is not readily available in many institutions and remains a highly invasive and complex procedure [15]. Another potential option is plasmapheresis. According to an editorial written on single-centre experience, patients poisoned with AIP who underwent plasmapheresis in less than six hours of aluminium phosphate poisoning had a better prognosis than those who presented later [16]. These results are still primitive, and accordingly, there is a need for clinical trials, comparative studies for better analysis of these elimination strategies.

Clinical Recommendations

**Figure 1:**
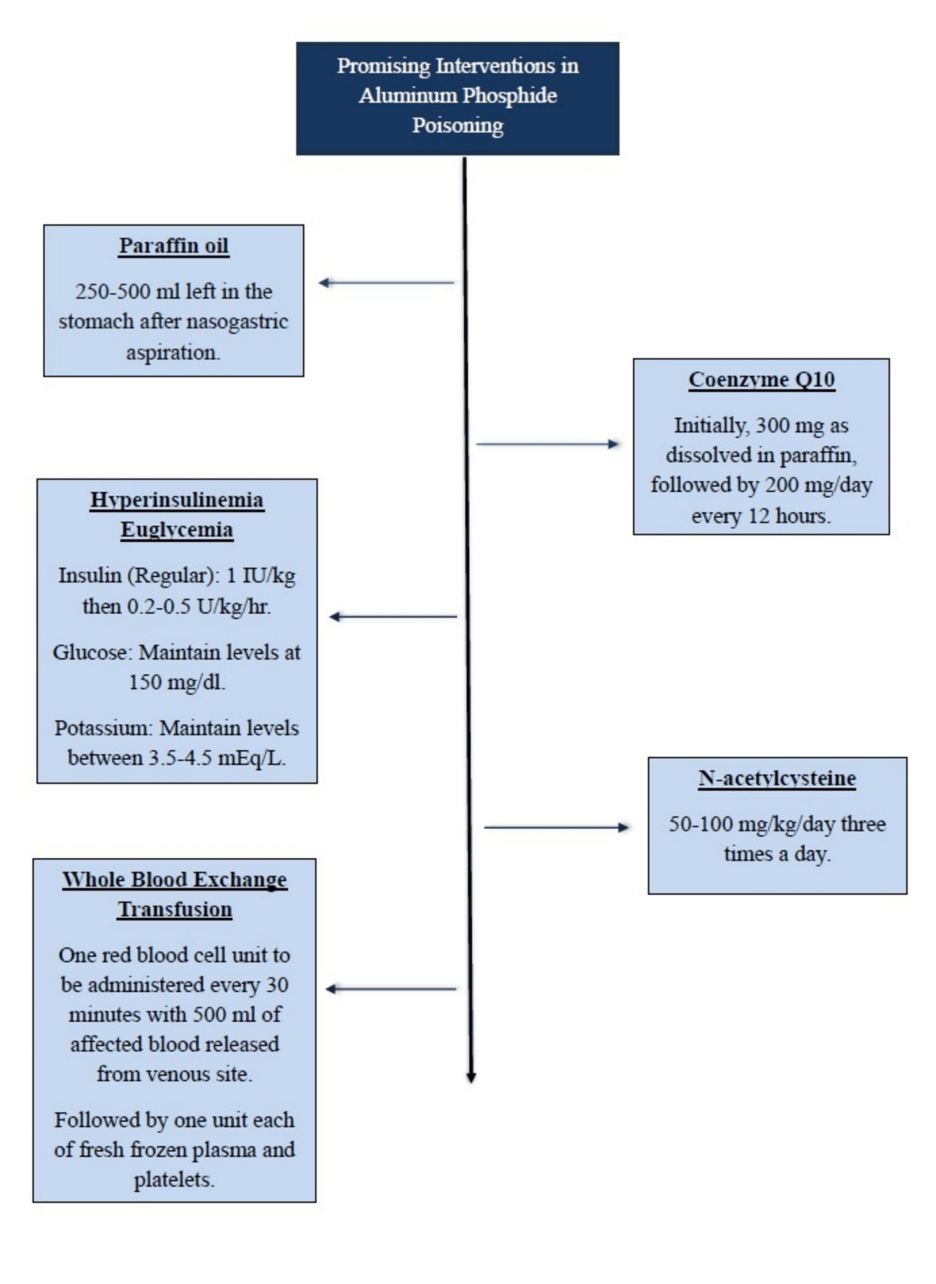
Author-proposed Treatment Recommendations for Aluminum Phosphide Poisoning.

Fluid therapy should be the mainstay for achieving hemodynamic stability to manage the persistent hypotension. Vasopressors can be used to augment the desired effects. β-receptor agonists like dopamine and dobutamine should be avoided because of the risk of arrhythmias. A trial of intravenous sodium bicarbonate therapy can be used to reverse metabolic acidosis before the initiation of hemodialysis. The patency of the airway should be ensured, and the patient should always be intubated before undergoing gastric lavage and gastric decontamination. Antioxidants play a pivotal role in reversing the effects of poisoning by minimising the formation of harmful reactive oxygen species [1, 2, 11-13]. The authors propose the recommendations in a graphical presentation after a meticulous literature review from the cited studies (Figure 1).

## Conclusions

Currently, there is no antidote available for acute AlP poisoning, and there is a lack of evidence-based standardized therapeutic regimen. Given the high mortality and increasing incidence of AlP poisoning, finding a modality that could reduce mortality is a dream of every clinician who deals with these cases. Fortunately, there is some evidence that gastric decontamination using paraffin oil and co-enzyme Q10, hyperinsulinemia euglycemia (HIE) therapy and N-acetyl cysteine (NAC) decreases mortality. NAC use has a moderate quality of evidence in a systematic review and meta-analysis. Nonetheless, it is a mortality-reducing option that should be translated into clinical practice. For the former two options, further large-scale randomized controlled trials are needed to definitively evaluate their mortality benefit. If found to be as promising as indicated, it would mean a world to the ones benefiting.
